# Comparison of the Oncological Outcomes Between Robot-Assisted and Abdominal Radical Hysterectomy for Cervical Cancer Based on the New FIGO 2018 Staging System: A Multicentre Retrospective Study

**DOI:** 10.3389/fonc.2022.879569

**Published:** 2022-06-30

**Authors:** Pengfei Li, Xuemei Zhan, Chifei Lv, Zhong Lin, Ying Yang, Wuliang Wang, Shaoguang Wang, Min Hao, Bin Zhu, Xiaonong Bin, Jinghe Lang, Ping Liu, Chunlin Chen

**Affiliations:** ^1^ Department of Obstetrics and Gynecology, Nanfang Hospital, Southern Medical University, Guangzhou, China; ^2^ Department of Gynecology, Jiangmen Central Hospital, Jiangmen, China; ^3^ Department of Gynaecology Liuzhou Maternity and Child Healthcare Hospital, Liuzhou, China; ^4^ Department of Obstetrics and Gynecology, Xinqiao Hospital, Army Medical University, Chongqing, China; ^5^ Department of Obstetrics and Gynecology, The Second Affiliated Hospital of Zhengzhou University, Zhengzhou, China; ^6^ Department of Gynecology, The Affiliated Yantai Yuhuangding Hospital of Qingdao University, Yantai, China; ^7^ Department of Gynecology, the Second Hospital of Shanxi Medical University, Taiyuan, China; ^8^ Department of Gynecology, The Affiliated Yiwu Women and Children Hospital of Hangzhou Medical College, Hangzhou, China; ^9^ Department of Epidemiology, College of Public Health, Guangzhou Medical University, Guangzhou, China; ^10^ Department of Obstetrics and Gynecology, Peking Union Medical College Hospital, Peking Union Medical College, Beijing, China

**Keywords:** cervical cancer, radical hysterectomy, robot-assisted surgery, laparotomy, oncological outcomes

## Abstract

**Objective:**

To compare the 3-year oncological outcomes of robot-assisted radical hysterectomy (RRH) and abdominal radical hysterectomy (ARH) for cervical cancer.

**Methods:**

Based on the clinical diagnosis and treatment for cervical cancer in the China database, patients with FIGO 2018 stage IA with lymphovascular space invasion (LVSI)-IB2 cervical cancer disease who underwent RRH and ARH from 2004 to 2018 were included. Kaplan–Meier survival analysis was used to compare the 3-year overall survival (OS) and disease-free survival (DFS) rate between patients receiving RRH and those receiving ARH. The Cox proportional hazards model and propensity score matching were used to estimate the surgical approach-specific survival.

**Results:**

A total of 1,137 patients with cervical cancer were enrolled in this study, including the RRH group (n = 468) and the ARH group (n = 669). The median follow-up time was 45 months (RRH group vs. ARH group: 24 vs. 60 months). Among the overall study population, there was no significant difference in 3-year OS and DFS between the RRH group and the ARH group (OS: 95.8% vs. 97.6% p = 0.244). The Cox proportional hazards analysis showed that RRH was not an independent risk factor for 3-year OS (HR: 1.394, 95% CI: 0.552–3.523, p = 0.482). However, RRH was an independent risk factor for 3-year DFS (HR: 1.985, 95% CI: 1.078–3.655 p = 0.028). After 1:1 propensity score matching, there was no significant difference in 3-year OS between the RRH group and the ARH group (96.6% vs. 98.0%, p = 0.470); however, the 3-year DFS of the RRH group was lower than that of the ARH group (91.0% vs. 96.1%, p = 0.025). The Cox proportional hazards analysis revealed that RRH was not an independent risk factor for 3-year OS (HR: 1.622, 95% CI: 0.449–5.860 p = 0.461), but RRH was an independent risk factor for 3-year DFS (HR: 2.498, 95% CI: 1.123–5.557 p = 0.025).

**Conclusion:**

Among patients with stage I A1 (LVSI +)-I B2 cervical cancer based on the FIGO 2018 staging system, RRH has a lower 3-year DFS than ARH, suggesting that RRH may not be suitable for early cervical cancer patients.

## Introduction

Robot-assisted surgery is becoming more and more common in gynaecological surgeries. However, abdominal radical hysterectomy (ARH) is still the standard treatment for cervical cancer according to the US National Comprehensive Cancer Network (NCCN) guidelines (1). The safety of robot-assisted radical hysterectomy in the treatment of cervical cancer is still controversial.

We previously reported the oncological outcomes of robot-assisted radical hysterectomy (RRH) and abdominal radical hysterectomy (ARH) for cervical cancer based on the Federation of Gynecology and Obstetrics (FIGO) 2009 staging system (2) and found that RRH was associated with worse 3-year oncological outcomes than ARH in patients with early-stage cervical cancer. In the FIGO 2018 staging system, patients with lymph node metastasis are classified as IIIC stage (3, 4). It is still unknown whether RRH is suitable for patients with FIGO 2018 stage IA with lymphovascular space invasion (LVSI)-IB2 cervical cancer disease.

Furthermore, as for the surgical approach of radical hysterectomy, most of the clinical studies cited in NCCN Clinical Practice Guidelines for cervical cancer are based on the FIGO 2009 staging system (5–8), and there is still a lack of clinical evidence based on the FIGO 2018 staging system. Therefore, it is necessary to compare the oncological outcomes of different surgical approaches based on the new FIGO 2018 staging system.

In this study, 1,137 patients with stage IA1 (LVSI+)~IB2 cervical cancer based on the new FIGO 2018 staging system were selected from the clinical diagnosis and treatment for cervical cancer in a mainland China database, and the 3-year oncological outcomes of RRH and ARH were compared.

## Materials and Methods

### The Data Source

This study was a multicentre, retrospective, cohort study, and the data of this study originated from the clinical diagnosis and treatment for cervical cancer in a mainland China database, a cervical cancer-specialized disease database (n = 63,926) that covers consecutive patients with cervical cancer in 47 hospitals in mainland China treated between January 2004 and December 2018. This retrospective study was approved by the Ethics Committee of Nanfang Hospital, Southern Medical University (ethics number NFEC-2017-135). The details of the data sources and methods were the same as previously reported (9–12).

Gynaecologists who were uniformly trained collected information on patients with cervical cancer who meet the eligibility requirements. The prognosis was followed up by gynaecologists who received unified training in each hospital. Follow-up was mainly conducted through outpatient services and telephone. This recorded the patients’ survival, recurrence and other information. To ensure the accuracy of data entry, two trained gynaecologists double-input the same case data and then set up a unified database after checking the doubtful parameters.

All cases in the database were re-staged according to the FIGO 2018 staging system. For example, the substage of stage IB was re-staged according to tumour size. Those with pathologically confirmed lymph node metastasis were classified as stage IIICp, and those with radiologically confirmed lymph node metastasis were classified as stage IIICr.

### Inclusion Criteria and Exclusion Criteria

The inclusion criteria were as follows: patients were ≥18 years of age or older. Patients received treatment in hospitals that can carry out both RRH and ARH. The cervical biopsy pathology was squamous adenocarcinoma and adenosquamous cervical cancer. The FIGO 2018 stages were stages IA1 (LVSI+), IA2, IB1, and IB2. No preoperative adjuvant treatment was administered. Surgical approaches were robot-assisted or open surgery. Surgery types were QM-B or QM-C radical hysterectomy + pelvic lymph node resection ± abdominal para-aortic lymph node resection.

The exclusion criteria were as follows: pregnant patients, cervical stump cancer, combined with other malignancies.

### Observational Index

The 3-year overall survival (OS) and disease-free survival (DFS) were observed. OS was defined as the date of diagnosis until death of any cause or the last effective follow-up. DFS was defined as the date of diagnosis until death/recurrence or the last valid follow-up date. For patients without recurrence and those who were still alive, the date of the last follow-up or date of the last outpatient visit was recorded.

### Statistical Methods

Measurement data are expressed as the mean ± standard deviation (x ± s), and classified data are expressed as percentages (%). Kaplan–Meier curves were used to describe the change in survival outcomes. Confounding factors were adjusted by the Cox proportional hazard regression model. The risk ratio and 95% confidence interval were used for estimating the effect of the surgical approach on 3-year OS and DFS rates, using SPSS 23.0 statistical software (SPSS, Inc., Chicago, IL, USA). p < 0.05 was considered statistically significant.

In the propensity score matching analysis (PSM), patients in the RRH group and the ARH group were matched according to the propensity score to reduce confounding bias and to create a new cohort of patients that underwent different surgical procedures but had similar clinicopathological features. Propensity score matching was performed for each patient and was calculated by a logical regression model, which included age, FIGO stage, tumour diameter, depth of cervical invasion, LVSI, parametrium metastasis, vaginal surgical margin and adjuvant therapy (10–13). The χ^2^ test was used to check whether the distribution of matching variables in the RRH group and ARH group was balanced.

## Results

### Case Screening Process and Results

**Figure 1:**
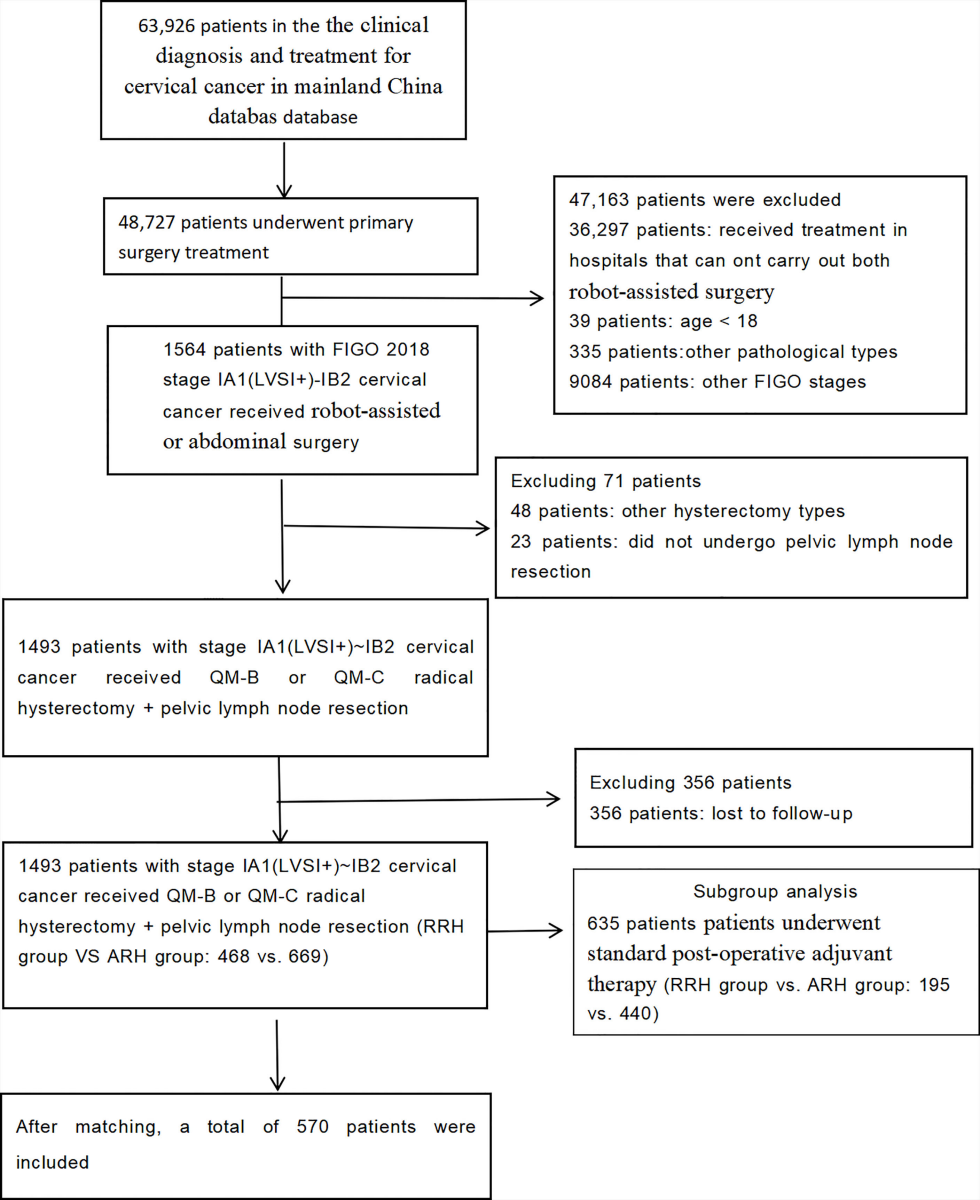
Flowchart of patients included in the analysis.

A total of 1,137 patients were included in the study. There were 468 patients in the RRH group and 669 patients in the ARH group. Their average age was 46.89 years. The median follow-up time was 43 months (RRH group vs. ARH group: 24 vs. 60 months). The patient selection process is shown in **Figure 1**.

### Clinicopathological Features of the Two Groups

The RRH was more likely to have deep interstitial invasion, LVSI, and positive vaginal margins than the ARH group. The RRH group was more likely to receive post-operative-assisted therapy than the ARH group was. The baseline differences of other clinicopathological characteristics were not statistically significant between the RRH and ARH groups, as shown in **Table 1**.

**Table 1:** The clinicopathologic characteristics of patients in the RRH group and ARH group.

Characteristics		Overall study population			After matching
	RRH n = 468 (%) ARH n = 669 (%)		p	RRH n = 285 (%) ARH n = 285 (%) p		
Age	47.01 ± 9.734	46.81 ± 9.334	0.172	46.65 ± 9.259	46.81 ± 8.724	0.273
FIGO stage			0.588			0.437
IA1 (LVSI+)	1 (0.1)	3 (0.1)		0	0	
IA2	17 (3.6)	33 (4. 9)		12 (4.2)	18 (6.3)	
IB1	190 (40.6)	285 (42.6)		130 (45.6)	120 (42.1)	
IB2	260 (55.6)	350 (52.3)		143 (50.2)	147 (51.6)	
Stromal invasion			<0.001			0.994
≤1/2	336 (71.8)	367 (54.9)		184 (64.6)	185 (64.9)	
>1/2	67 (14.3)	235 (35.1)		55 (19.3)	54 (18.9)	
Unknown	65 (13.9)	67 (10.0)		46 (16.1)	46 (16.1)	
LVSI			0.004			0.614
Negative	382 (81.6)	587 (87.7)		251 (88.1)	247 (86.7)	
Positive	86 (18.4)	82 (12.3)		34 (11.9)	38 (13.3)	
Parametrium			0.147			——
Negative	468 (100.0)	666 (99.6)		285 (100.0)	285 (100.0)	
Positive	0 (0.0)	3 (0.4)		——	——	
Vaginal margin			0.003			——
Negative	462 (98.7)	669 (100.0)		285 (100.0)	285 (100.0)	
Positive	6 (1.3)	0 (0.0)		——	——	
Tumour size			0.403			0.400
≤2	193 (41.2)	292 (43.6)		132 (46.3)	122 (42.8)	
>2	261 (55.8)	350 (52.3)		143 (50.2)	147 (51.6)	
Unknown	14 (3.0)	27 (4.0)		10 (3.5)	16 (5.6)	
Post-operative adjuvant Therapy			<0.001			0.802
None	179 (38.2)	432 (64.6)		156 (54.7)	160 (56.1)	
Chemotherapy	48 (10.3)	82 (12.3)		35 (12.3)	30 (10.5)	
Radiochemotherapy/radiotherapy	241 (51.5)	155 (23.2)		94 (33.0)	95 (33.3)	

### Survival Analysis in the Overall Study Population

**Figure 2:**
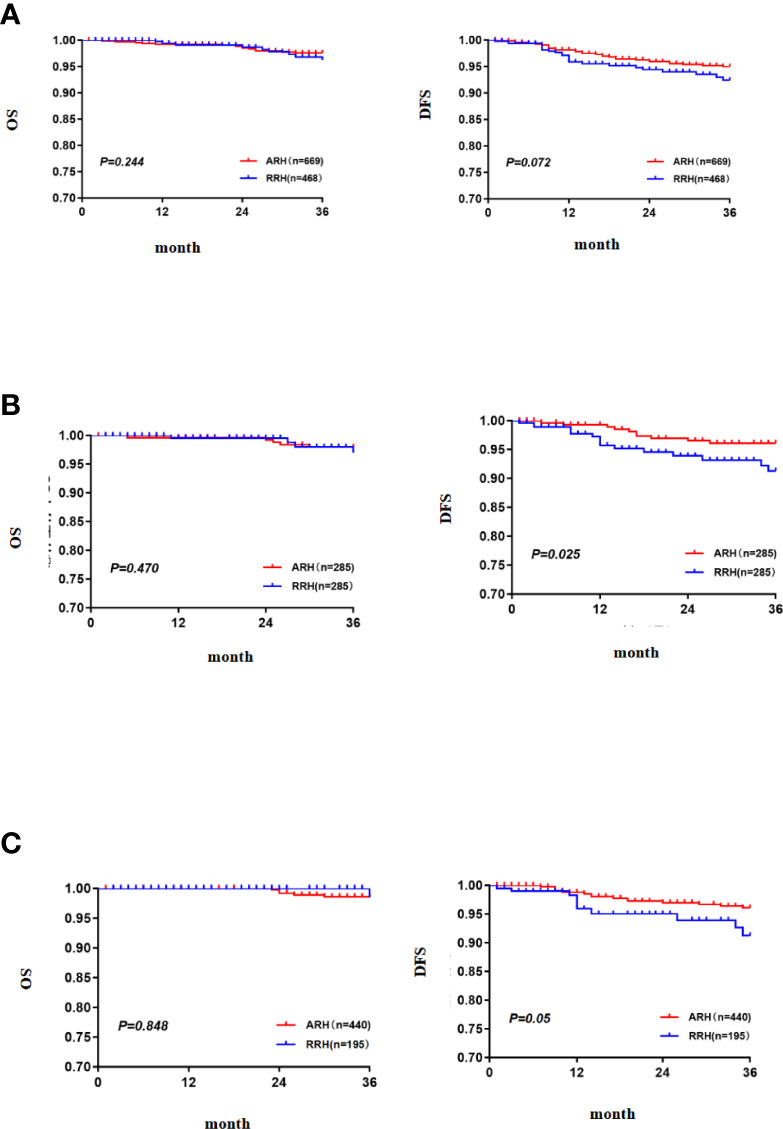
The 3-year OS and DFS [**(A)** the 3-year OS and DFS in the total study population, **(B)** the 3-year OS and DFS after PSM, **(C)** the 3-year OS and DFS in the subgroup of standard post-operative adjuvant therapy].

In the overall study population, the 3-year OS of the patients with FIGO 2018 stages IA1 (LVSI+)~IB2 cervical cancer in the RRH group was 95.8% and that of those patients in the ARH group was 97.6%. There was no significant difference between the two groups (p = 0.244) (**Figure 2A**). Cox multivariate analysis was used to control variables such as age, FIGO stage, tumour diameter, depth of cervical invasion, LVSI, parauterine metastasis, vaginal stump infiltration and post-operative assisted therapy. The results showed that RRH was not an independent risk factor for 3-year OS (HR: 1.394, 95% CI: 0.552–3.523, p = 0.482) (**Table 2**). The 3-year DFS of the patients with stage IA1 (LVSI+)~IB2 cervical cancer in the RRH group and ARH group was 92.0% and 95.0%, respectively. There was no significant difference in the 3-year DFS observed (p= 0.072) (**Figure 2A**). However, according to the Cox multivariate analysis, RRH was an independent risk factor for 3-year DFS (HR: 1.985, 95% CI: 1.078–3.655, p = 0.028) (**Table 2**).

**Table 2:** Cox multivariate analysis of the relationship between the surgical approach and OS and DFS in cervical cancer.

Characteristics		Multivariate analysis of 3-year OS		Multivariate analysis of 3-year DFS		
	HR	95% CI	p	HR	95% CI	p
Age	1.006	0.978–1.035	0.692	1.001	0.983–1.019	0.926
FIGO stage						
IA1 (LVSI+)	1.035	0.000–3.090E+44	0.999	1.005	0.977–1.033	0.742
IA2	1 (ref)	–	–	1(Ref)	–	–
IB1	34.758	0.000–7.287E+15	0.833	123.668	0.000–1.514E+18	0.799
IB2	279.814	0.000–1.069E+89	0.956	711.016	0.000–3.539E+76	0.940
Stromal invasion						
≤1/2	1 (ref)	–	–	1 (ref)	–	–
>1/2	1.577	0.624–3.990	1.577	2.044	1.115–3.747	0.021
Unknown	0.526	0.068–4.086	0.539	0.369	0.087–1.564	0.176
LVSI						
Negative	1 (ref)	–	–	1 (ref)	–	–
Positive	1.280	0.469–3.490	0.967	1.417	0.714–2.812	0.319
Parametrium						
Negative	1 (ref)	–	–	1 (ref)	–	–
positive	0.010	0.000–3.559E+21	0.868	0.004	0.000–6.807E+23	0.858
Vaginal margin						
Negative	1 (ref)	–	–	1 (ref)	–	–
Positive	0.007	0.000–7.599E+19	0.847	0.003	0.000–2.119E+20	0.832
Tumour size						
≤2	1 (ref)	–	–	1 (ref)	–	–
>2	0.986	0.000–4.465E+84	0.986	0.258	0.000–2.148E+71	0.987
Unknown	0.985	0.000–2.870E+16	0.985	1.677	0.000–1.635E+18	0.980
Post-operative adjuvant therapy						
None	1 (ref)	–	–	1 (ref)	–	–
Chemotherapy	1.565	0.390–6.284	0.528	0.612	0.227–1.653	0.333
Radiochemotherapy/radiotherapy	2.499	0.925–6.756	0.071	0.950	0.510–1.768	0.871
Surgical approach						
Laparotomy	1 (ref)	–	–	1 (ref)	–	–
Robot-assisted surgery	1.394	0.552–3.523	0.482	1.985	1.078–3.655	0.028

### Survival Analysis After Matching

After 1:1 PSM, there were 285 cases in each group. The table of baseline characteristics showed that the matching effectively reduced the confounding bias in the clinicopathological variables between the RRH group and ARH group (**Table 1**). The 3-year OS in the RRH group was 98.0%, and that in the ARH group was 96.6%. There was no significant difference between the two groups (p = 0.470) (**Figure 2B**). After adjusting for mixed cases by Cox multivariate analysis, RRH was not found to be an independent risk factor for 3-year OS (HR: 1.622, 95% CI: 0.449–5.860, p = 0.461). The 3-year DFS of the RRH group was lower than that of the ARH group (91.0% vs. 96.1%, p = 0.025, as shown in **Figure 2B**). The Cox multivariate analysis revealed that robotic surgery was found to be an independent risk factor for 3-year DFS (HR: 2.498, 95% CI: 1.123–5.557, p = 0.025, see **Table 3**).

**Table 3:** Cox multivariate analysis of the relationship between surgical approach and OS and DFS in cervical cancer after PSM.

Feature		3-year OS COX		3-year DFS COX		
	HR	95% CI	p	HR	95% CI	p
Age	0.949	0.881–1.021	0.163	0.970	0.929–1.014	0.180
FIGO stage						
IA2	1 (ref)	–	–	1 (ref)	–	–
IB1	484.563	0.000–1.587E+111	0.961	2.664.987	0.000–2.512E+107	0.949
IB2	1316.361	0.000–4.303E+111	0.955	5814.695	0.000–5.479E+107	0.943
Depth of cervical infiltration						
≤1/2	1 (ref)	–	–	1 (ref)	–	–
>1/2	0.887	0.167–4.710	0.888	2.419	0.951–6.153	0.064
Unknown	1.154	0.127–10.530	0.899	0.563	0.125–2.535	0.454
LVSI						
Negative	1 (ref)	–	–	1 (ref)	–	–
Positive	1.911	0.370–9.867	0.440	1.311	0.430–3.999	0.634
Parametrium						
Negative	1 (ref)	–	–	1 (ref)	–	–
Positive	0			0		
Vaginal margin						
Negative	1 (ref)	–	–	1 (ref)	–	–
Positive	0			0.000	0.000–5.236E+193	0.969
Tumour diameter						
≤2	1 (ref)	–	–	1 (ref)	–	–
>2	–	–	–	–	–	–
Unknown	0.779	0.000–1.233E+121	0.999	1.721	0.000–2.867E+115	0.997
Post-operative adjuvant therapy						
None	1 (ref)	–	–	1 (ref)	–	–
Chemotherapy	2.791	0.437–17.835	0.278	0.658	0.176–2.465	0.535
Radiochemotherapy/radiotherapy	2.144	0.431–10.672	0.351	0.663	0.257–1.709	0.395
Surgical approach						
Laparotomy	1 (ref)	–	–	1 (ref)	–	–
Robot-assisted surgery	1.622	0.449–5.860	0.461	2.498	1.123–5.557	0.025

### Survival Analysis in the Subgroup of Standard Post-Operative Adjuvant Therapy

For patients who underwent standard post-operative adjuvant therapy, there was no significant difference in 3-year OS and DFS between the RRH (195 cases) and ARH (440 cases) groups (98.5% vs. 98.6% p = 0.848; 91.2% vs. 96.1% p = 0.05, as shown in **Figure 2C**). After the confounding factors were eliminated by Cox multivariate analysis, RRH was not found to be an independent risk factor for 3-year OS (HR: 1.065, 95% CI: 0.112–10.105, p = 0.956). However, RRH was an independent risk factor for 3-year DFS (HR: 3.329, 95% CI: 1.379–8.039, p = 0.007).

## Discussion

The study screened 1,137 patients with FIGO 2018 stage IA1 (LVSI+)-IB2 and underwent RRH and ARH from the clinical diagnosis and treatment for cervical cancer in the mainland China database. We compared the 3-year oncological outcomes of RRH and ARH for FIGO 2018 stage IA (LVSI+)-IB2 cervical cancer, and we found that the 3-year OS and DFS of RRH and ARH were similar in the total study population. The multivariable analysis results revealed that RRH was not an independent risk factor affecting 3-year OS, but RRH was an independent risk factor affecting 3-year DFS. After propensity score matching, the results obtained were the same as those before matching, suggesting that RRH was not suitable for FIGO 2018 stage IA1 (LVSI+)-IB2 cervical cancer patients. To eliminate the influence of standard adjuvant treatment on the study results, a subgroup analysis of patients who received standard post-operative adjuvant therapy was also conducted, and the same conclusion was obtained, further suggesting that RRH is not suitable for FIGO 2018 stage IA1 (LVSI+)-IB2 cervical cancer patients.

Furthermore, previous studies comparing oncological outcomes between RRH and ARH were based on the FIGO 2009 staging system. The study of Ohlmann et al. included 631 patients with FIGO 2009 cervical cancer and found that both laparoscopic surgery and RRH resulted in worse DFS than ARH (14). Chen et al. showed that the 3-year OS of patients receiving RRH was similar to that of patients receiving ARH, but the 3-year DFS of patients receiving RRH was lower than that of patients receiving ARH (13). The study by Benny Brandt et al. exploring minimally invasive surgery and ARH in patients with stage IA1 (LVSI+)-IB1 cervical cancer showed that minimally invasive surgery was similar to ARH of OS and DFS (15). The study by Chiva et al. investigating minimally invasive surgery and ARH in patients with stage IB1 cervical cancer showed that minimally invasive surgery increased the risk of relapse and death in patients with early cervical cancer in a European population (16). There was a 10% difference in the 4.5-year disease-free survival between the two surgical approaches, which was similar to the DFS findings in this study. By comparing the oncological outcomes of early cervical cancer patients receiving ARH and those receiving minimally invasive surgery, Kim et al. found that the minimally invasive surgery group displayed poorer progression-free survival. Multivariate analyses identified minimally invasive surgery as an independent poor prognostic factor for progression-free survival (17). However, all the above studies were based on the FIGO 2009 staging system. This multicentre study is the first to compare the oncological outcomes of RRH and ARH based on the FIGO 2018 staging system.

Both this study and previous studies based on the FIGO 2009 system suggest that the prognosis of robot-assisted minimally invasive surgery is worse than that of open surgery, which may be related to the fact that RRH is more likely to cause tumour dissemination than ARH. Tumour tissue may adhere to RRH instruments and thus be transferred to the next surgical site, leading to metastasis. Compared with the study by Chen et al., the new FIGO 2018 staging system was adopted for the inclusion criteria in this study, while FIGO 2009 was adopted for the study by Chen et al. There was no statistically significant difference in OS between the two surgical approaches, which may be due to the implementation of post-operative adjuvant therapy. The DFS was lower in the RRH group than in the ARH group, but the proportion of patients in the RRH group receiving post-operative adjuvant therapy was higher than that in the ARH group, indicating that post-operative adjuvant therapy was conducive to prolonging the survival time of patients. Differences in post-operative adjuvant therapy may also affect the results, so in this study, post-operative adjuvant therapy was also included in the Cox multivariate analysis to eliminate the interference of confounding factors. Subgroup analysis was performed on the standard of adjuvant treatment after surgery.

There are some limitations to this study. First, RRH was adopted late in Chinese mainland, and only a few patients were followed for 5 years. The use of 3-year OS and DFS rates in this study may lead to bias in the results. Second, the experience level of the surgeon can affect the post-operative oncological outcome to a certain extent, but the learning curve of surgeons for the two kinds of surgery was not included in this study (3, 9, 18). Third, this study was a retrospective study, and although the influence of known confounding factors on the results could be reduced by the multivariate analysis and propensity score matching, the confounding factors could not be completely eliminated. More randomized controlled trials are needed to the oncologic safety of RRH for early-stage cervical cancer. The results of the robot-assisted approach to cervical cancer (RACC) are expected; this trial is an ongoing prospective, multi-institutional, international, open-label randomized clinical study (19). The aims of this study is to compare the recurrence-free survival at 5 years between women who underwent robot-assisted laparoscopic surgery versus laparotomy for early-stage cervical cancer.

## Conclusion

In conclusion, RRH and ARH showed similar 3-year OS in early cervical cancer patients with FIGO 2018 stage IA1 (LVSI+)~IB2 disease, but the 3-year DFS of RRH was lower than that of ARH. This multicentre, large-sample retrospective study demonstrates that RRH is not suitable for the treatment of early cervical cancer patients based on the FIGO 2018 staging system.

## Data Availability Statement

The datasets generated for this study are available on request to the corresponding authors.

## Author Contributions

PL, XZ, and CL contributed equally to the work; PeL: literature search, data collection, data analysis and interpretation, methodology, writing-original draft, writing-review and editing; XZ: data curation, writing-original draft, writing-review and editing; CL: literature search, data collection, investigation, writing—review and editing; ZL: investigation, writing—original draft; YY: investigation, resources; WW: investigation, resources; SW: investigation, resources; MH: investigation, resources; XZ: investigation, resources; BZ: investigation, resources; XB: investigation, resources; JL: supervision, conceptualization; PiL: supervision, conceptualization, project administration, writing—review and editing; CC: supervision, conceptualization, project administration, funding acquisition, writing—review and editing. All authors contributed to the article and approved the submitted version.

## Funding

This study was supported by the National Science and Technology Support Program of China (2014BAI05B03), the National Natural Science Fund of Guangdong (2015A030311024), and the Science and Technology Plan of Guangzhou (158100075).

## Conflict of Interest

The authors declare that the research was conducted in the absence of any commercial or financial relationships that could be construed as a potential conflict of interest.

## Publisher’s Note

All claims expressed in this article are solely those of the authors and do not necessarily represent those of their affiliated organizations, or those of the publisher, the editors and the reviewers. Any product that may be evaluated in this article, or claim that may be made by its manufacturer, is not guaranteed or endorsed by the publisher.
